# Enhanced recovery after surgery (ERAS) in adolescent idiopathic scoliosis (AIS): a meta-analysis and systematic review

**DOI:** 10.1007/s43390-021-00310-w

**Published:** 2021-03-16

**Authors:** Akshay D. Gadiya, Jonathan E. J. Koch, Mohammed Shakil Patel, Masood Shafafy, Michael P. Grevitt, Nasir A. Quraishi

**Affiliations:** Centre for Spinal Studies and Surgery, D Floor, West Block, Queens Medical Centre, Derby Road, Nottingham, NG7 2UH UK

**Keywords:** Enhanced recovery after surgery, Fast-track recovery, Adolescent idiopathic scoliosis, Posterior spinal fusion

## Abstract

**Study design:**

A systematic review reporting on the efficacy of an ERAS protocol in patients undergoing spinal fusion for AIS.

**Objective:**

To systematically evaluate the relevant literature pertaining to the efficacy of ERAS protocols with respect to the length of stay, complication, and readmission rates in patients undergoing posterior spinal corrective surgery for AIS.

**Summary of background data:**

ERAS is a multidisciplinary approach aimed at improving outcomes of surgery by a specific evidence-based protocol. The rationale of this rapid recovery regimen is to maintain homeostasis so as to reduce the postoperative stress response and pain. No thorough review of available information for its use in AIS has been published.

**Methods:**

A systematic review of the English language literature was undertaken using search criteria (postoperative recovery AND adolescent idiopathic scoliosis) using the PRISMA guidelines (Jan 1999-May 2020). Isolated case reports and case series with < 5 patients were excluded. Length of stay (LOS), complication and readmission rates were used as outcome measures. Statistical analysis was done using the random effects model.

**Results:**

Of a total of 24 articles, 10 studies met the inclusion criteria (9 were Level III and 1 of level IV evidence) and were analyzed. Overall, 1040 patients underwent an ERAS-type protocol following posterior correction of scoliosis and were compared to 959 patients following traditional protocols. There was a significant reduction in the length of stay in patients undergoing ERAS when compared to traditional protocols (*p* < 0.00001). There was no significant difference in the complication (*p* = 0.19) or readmission rates (*p* = 0.30). Each protocol employed a multidisciplinary approach focusing on optimal pain management, nursing care, and physiotherapy.

**Conclusion:**

This systematic review demonstrates advantages with ERAS protocols by significantly reducing the length of stay without increasing the complications or readmission rates as compared to conventional protocols. However, current literature on ERAS in AIS is restricted largely to retrospective studies with non-randomized data, and initial cohort studies lacking formal control groups.

**Level of evidence:**

3.

## Introduction

Corrective surgery for Adolescent Idiopathic Scoliosis (AIS), involves many perioperative challenges including patient and family counseling, optimization of the patient, adequate pain control, effective management of side-effects due to opioids, and early mobilization amongst others [1]. If these are not appropriately addressed, then they may result in a delayed return to function, prolonged hospital stay, and increased costs.

Enhanced recovery after surgery (ERAS) is a multimodal and multidisciplinary approach for improving perioperative outcomes of patients using sub-specialty specific evidence-based protocols in the care of the surgical patient [2]. To-date, many studies across a range of specialties have highlighted the benefits of ERAS in reducing the length of stay (LOS) and improving the outcomes following surgery [3–7]. Moreover, ERAS has also been shown to have a positive financial impact on healthcare management [8].

A recently published narrative review has reported on the efficacy of early mobilization in spinal surgery in improving postoperative recovery and outcomes [9]. Goal directed-early mobilization using evidence-based protocols improve patient-reported outcomes as compared to bed rest after elective spine surgery [9]. Despite there being a lack of consensus on the most effective protocol, mobilization on the same day following elective spinal surgery can be achieved [9]. A recent systematic review reported a reduction in the length of stay without an increase in complications or readmission rates following the use of ERAS-based protocols after elective spine surgery [3]. This study, however, was not specific to patients undergoing posterior scoliosis correction.

Posterior correction of idiopathic scoliosis is an extensive surgical procedure for an adolescent patient. Sub-optimal control of postoperative pain results in delayed mobilization. Mobility is also affected due to the morbidity associated with nausea, vomiting, sedation and ileus as a result of the use of intravenous opioid analgesia [1]. This delayed mobilization results in increased length of stay and increased complications [9] as well as an increased financial burden to the healthcare systems.

The implementation of an ERAS based protocol is aimed to expedite the recovery and return of function, minimize the morbidity and in turn reduce the LOS associated with the PSF in patients with AIS. Reduction in time spent in hospital would also improve the over-all peri-operative experience of patients and reduce health care costs [13].

The aim of this study was to perform a systematic review and meta-analysis of the literature evaluating studies implementing ERAS protocols for the length of stay, complication rates, and readmission rates in patients undergoing posterior corrective surgery for AIS.

## Methods

### Data sources

A literature search was performed using PUBMED and MEDLINE databases for articles published between December 1999 to May 2020 using “adolescent idiopathic scoliosis”, “idiopathic scoliosis”, “scoliosis”, “deformity correction”, “posterior spinal fusion”, “accelerated recovery protocol”, “discharge protocol”, “pain management”, “postoperative recovery”, “accelerated discharge”, “hospital stay” and “complications” as keywords in combination with Boolean “AND” and “OR” phrases. We used “postoperative recovery AND adolescent idiopathic scoliosis” as search criteria.

Titles and abstracts were screened for importance and relevance, and articles describing the implementation of enhanced-recovery protocols with regards to posterior correction for adolescent idiopathic scoliosis were included. References of literature included in the final qualitative and quantitative analyses were searched manually for additional relevant studies that were not found in the initial search.

### Selection criteria

**Fig. 1:**
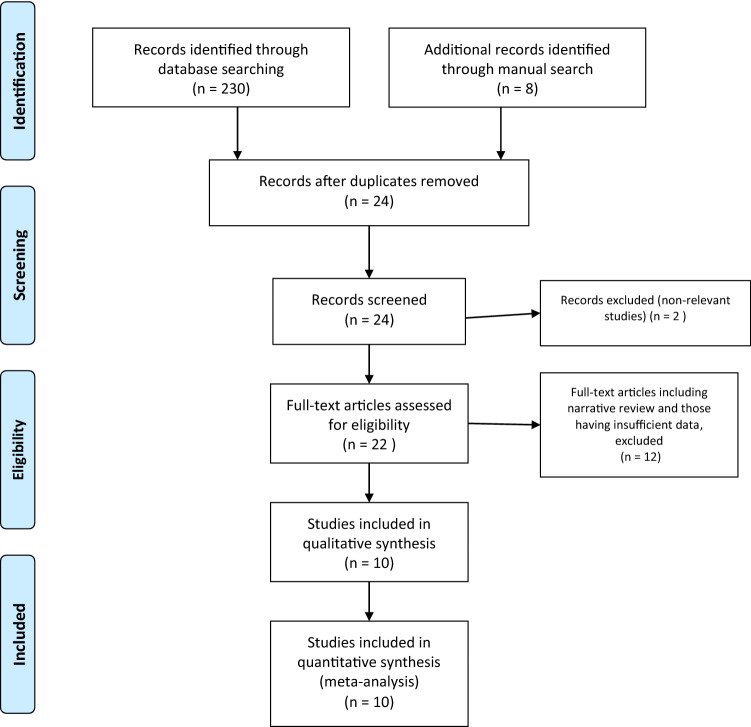
PRISMA chart for systematic review of database

**Fig. 2:**
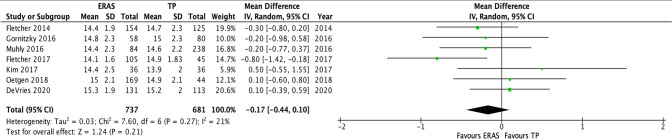
Analysis of age

Selected articles included adolescent patients with idiopathic scoliosis undergoing posterior correction surgery. These selected articles were reviewed by two authors (ADG and JEJK). Included articles had to report on the efficacy of rapid recovery protocols. Case reports, expert opinions, narrative reviews and studies comparing and evaluating the efficacy of various modes of analgesia in isolation were excluded. Since ERAS protocols are intended to address various factors involved in the perioperative management of patients, articles evaluating the efficacy of a single variable in the outcome of patients undergoing PSF for AIS were excluded. Figures 1 and 2 demonstrates the flowchart of the methodology. Outcomes assessed were the length of stay (LOS), rate of readmissions and rate of primary complications. The operative duration, estimated blood loss, preoperative cobb angles and the number of levels fused were also compared to ensure that there was no significant differences between the two groups.

### Data extraction

Using a data extraction form, two investigators (ADG and JEJK) independently evaluated all the studies and summarized the following information: (1) study characteristics (year of publication, design and recruitment period); (2) patient characteristic (number of patients, demographics and clinical characteristics); (3) eligibility (based on above mentioned study selection criteria); (4) perioperative protocols utilized to enhance the recovery; (5) outcomes measures as described previously.

After summarizing the data, any discrepancy between the two investigators was identified and if needed the opinion of a third investigator (MSP, MPG or NAQ) was sought to settle the differences. Both primary investigators, for quality and accuracy again screened extracted data.

### Statistical methods

The Meta-analysis was conducted using Cochrane Review Manager RevMan 5.3. The mean and standard deviations for the length of stay were compared between the two groups. Data from studies which were presented in the form of median and interquartile ranges were converted to estimated means and standard deviations by the methods demonstrated by Luo et al. [10] and Wan et al. [11]. Studies that did not include any values for dispersion from the mean could not be analysed and were therefore excluded. The inverse variance method of analysis to assess LOS was used. The Mantel–Haenszel method was used to calculate the Odds ratios and 95% CIs for assessment of the complications and readmission rates. The inverse variance method of analysis to assess the blood loss, age, operative time, preoperative Cobb angles, and the number of levels fused was also assessed to ensure there were no overall significant differences between the two groups. Studies with zero events in both arms were excluded. Due to the variation in the ERAS and TP protocols between studies, a random effect model for statistical analysis was used for all outcomes. In the overall effect *Z* test, a *p* value < 0.05 was deemed statistically significant. Heterogeneity was evaluated using the *I*^2^ statistic.

## Results

Of a total of 21 articles, 10 studies met the inclusion criteria (9 of Level III and 1 of level IV evidence). These studies were subjected to quantitative and qualitative analysis for evaluating the outcomes. Overall, 1040 patients were identified who underwent an ERAS-type protocol peri-operatively during posterior correction of scoliosis and were compared to 959 patients who underwent conventional protocols. Table 1 illustrates the data from all included studies.

**Table 1:** Results of systematic review of ERAS in AIS

SR	Author	Design	Level of evidence	*N*		LOS (days)		Complication rate (%)		Readmission rate (%)	
				ERAS	TP	ERAS	TP	ERAS	TP	ERAS	TP
1	Fletcher et al. [12]	Retrospective	Level III	154	125	2.92 ± 0.71	4.28 ± 1.08	15.6 (24/154)	10.4 (13/125)	NA	NA
2	Muhly et al. [13]	Retrospective	Level III	84	238	4	5.7	NA	NA	3.6 (3/84)	2.9 (7/238)
3	Gornitzky et al. [14]	Retrospective	Level III	58	80	3.5 ± 0.8	5.0 ± 0.8	NA	NA	5 (3/58)	3 (2/80)
4	Sanders et al. [15]	Retrospective	Level III	90	194	3.7 ± 0.93	5.0 ± 1.26	5.55 (5/90)	12.89 (25/194)	4.4 (4/90)	1.03 (2/194)
5	Rao et al. [16]	Retrospective	Level III	139	51	4.1 ± 1.16	NA	1.4 (2/139	12 (6/51)	NA	NA
						3.5 ± 1.13				NA	
6	Fletcher et al. [17]	Retrospective	Level III	105	45	2.17 ± 0.314	4.21 ± 1.51	7.6 (8/105)	20 (9/45)	NA	NA
7	Chan et al. [18]	Prospective	Level IV	74	33	3.6 ± 0.6	5.23 ± 2.43	NA	NA	NA	NA
8	Kim et al. [19]	Prospective	Level III	36	36	3.3 ± 0.6	3.9 ± 0.7	2.7 (1/36)	2.7 (1/36)	0	0
9	Oetgen et al. [20]	Retrospective	Level III	169	44	NA	5.1	2.36 (4/169)	2.27 (1/44)	8.28 (14/169)	9.1 (4/44)
10	DeVries et al. [21]	Retrospective	Level III	131	113	3.4	5.2	5.34 (7/131)	4.42 (5/113)	12.9 (17/131)	11.5 (13/113)

### Age

All 10 studies presented data on the average age of patients. Sanders et al. did not provide any measures of dispersion from the mean and was excluded. Rao et al. only provided the average age with the age range and was again excluded. Chan et al. did not provide the average age for the TP group and again was excluded. This left 7 studies for inclusion in the meta-analysis. A total of 737 and 681 patients were analyzed for the ERAS and TP respectively with regards to age. The analysis demonstrated there not to be any significant difference in the ages between the studies, 95% CI − 0.44 to 0.1, *p* = 0.21. There was small amount of heterogeneity between the studies with *I*^2^ = 21%, which was statistically insignificant, *p* = 0.27 and is illustrated in Figs. 1 and 2.

### Operative time

**Fig. 3:**
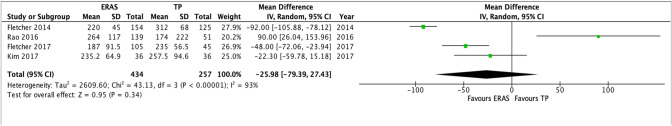
Analysis of operative time

6 studies presented data on the operative time. Sanders et al. did not provide any data on the dispersion from the mean and Chan did not present the data for the TP group leaving 4 studies which were included in the analysis. The operative time for a total of 434 patients was compared to 257 patients in the ERAS and TP groups respectively. Although individually 2 studies demonstrated operative time to be shorter in the ERAS group and 1 study for the TP group, overall there was no significant difference in the operative time when the data was pooled together with a mean difference of 25.98 min, 95% CI − 79.39 to 27.43, *p* = 0.34. As would be expected, there was significant heterogeneity between the studies, with *I*^2^ = 93%, *p* < 0.00001 and is illustrated in Fig. 3.

### Number of levels fused

**Fig. 4:**
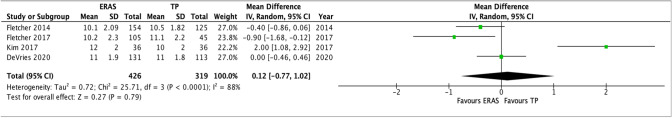
Analysis of number of levels fused

5 studies presented data on the number of levels fused. Sanders et al. again did not present any measures for dispersion from the mean and was therefore excluded leaving 4 studies included in the metanalysis. Overall, 426 patients in the ERAS group were compared to 319 patients in the TP group demonstrating there to not be a significant difference in the number of levels fused between the two groups, 95% CI − 0.77 to 102, *p* = 0.79. There was significant heterogeneity between the groups with *I*^2^ = 88%, *p* < 0.0001 and is illustrated in Fig. 4.

### Preoperative magnitude of main curve

**Fig. 5:**
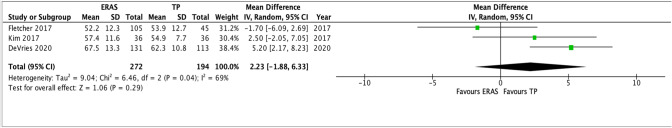
Analysis of preoperative magnitude of main curve

6 studies presented data on the preoperative Cobb angles. Again, Sanders et al. did not present any measures of dispersion from the mean, Chan et al. did not provide this data for the TP group, and Fletcher et al. only provided the mean with the range, and therefore, these three studies were excluded leaving only three studies for inclusion in the meta-analysis. No data was available on the post op Cobb angles. The meta-analysis demonstrated there to be no significant difference between the groups in terms of preop Cobb angles, MD 2.23, 95% CI − 1.88 to 6.33, *p* = 0.29. There was moderate amount of heterogeneity between the groups with *I*^2^ = 69%, *p* = 0.04 and is illustrated in Fig. 5.

### Blood loss

**Fig. 6:**
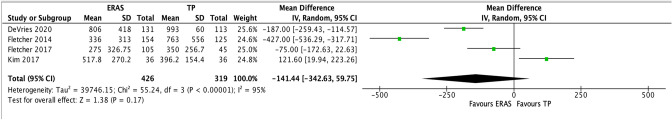
Analysis of blood loss

4 studies presented data on the intraoperative blood loss, allowing for 426 patients in the ERAS group to be compared to 319 patients in the TP group. The pooled data demonstrated there to be no significant differences between the two groups with a mean difference of − 141.44 ml, 95% CI − 342.6 to 59.75, *p* = 0.17. There was significantly large heterogeneity between the two groups with *I*^2^ = 95%, *p* < 0.00001 and is illustrated in Fig. 6.

### Length of stay

**Fig. 7:**
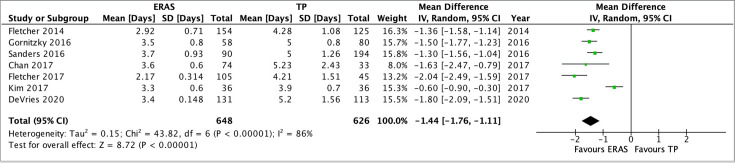
Analysis of length of stay

Eight studies presented data on the length of stay. One of these study’s [13] was excluded as they did not present measures of dispersion from the mean. Seven studies were therefore included in the meta-analysis A total of 648 patients having had Enhanced recovery and 626 with the traditional protocols (TPs). Individually, all studies demonstrated a significant reduction in the LOS with an ERAS protocol as compared to the traditional methods. The meta-analysis demonstrated an ERAS protocol to significantly reduce the length of stay by an average of 1.44 days (95% CI − 1.11 to 1.76, *p* < 0.00001). There was significant heterogeneity between the studies with *I*^2^ = 86%, *p* < 0.00001, and is illustrated in Fig. 7.

### Complication rate

**Fig. 8:**
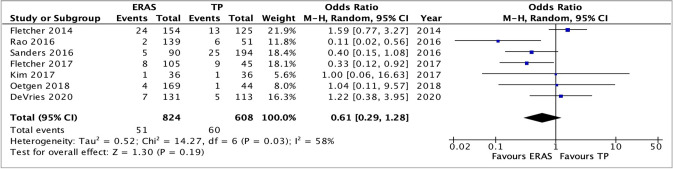
Analysis of complication rate

Five studies compared the complications rates between ERAS and TPs with a total of 524 patients and 451 patients respectively. Pooling the data together, did not demonstrate any significant difference in complications between the two groups (OR 0.48, 95% CI 0.18–1.28, *p* = 0.14). There was moderate heterogeneity between the groups, *I*^2^ = 70%, *p* = 0.01 (Fig. 8).

### Readmission rate

**Fig. 9:**
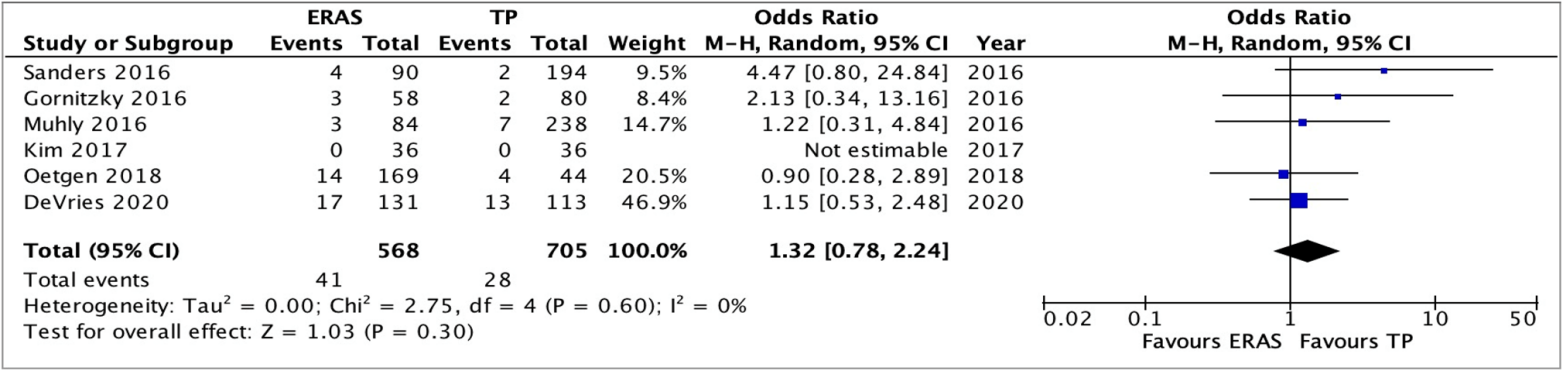
Analysis of readmission rate

Six studies compared the readmission rates between the two recovery protocols. One study [19] reported zero readmissions in both groups and was therefore excluded. This left a total of 532 patients in the ERAS group and 669 in the TP group. Overall, pooling the data did not demonstrate any significant increase in the readmission rates between patients having an ERAS or TP pathways, OR 1.32, 95% CI 0.78–2.24, *p* = 0.30. There was no heterogeneity between the groups with *I*^2^ = 0%, *p* = 0.60 (Fig. 9).

### Qualitative analysis

All 10 studies placed great emphasis on patient-centered optimization strategies to improve the outcomes following corrective surgery. Though there were minor differences in protocols, the common aim was to reduce the stress response, surgery related pain and maintain homeostasis following surgery. All studies proposed the involvement of a multidisciplinary team for executing their protocol. Table 2 illustrates the recommended ERAS protocol based on this systematic review. The ERAS protocols utilized in all these studies can be summarized under the following three headings:

**Table 2:** Summary of qualitative analysis of the utilization of ERAS protocols in AIS

Preoperative	Intraoperative	Postoperative
Open and transparent communication between patient/family and carers Patient education regarding anticipated recovery Early involvement of a spinal physiotherapist Use of oral heamatinics and multivitamins Chlorhexidine bath before surgery	Specific anaesthesia protocol IV tranexamic acid during surgery Use of cell Salvage techniques Isovolaemic hemodilution if appropriate Dual attending consultant Limited use of fluoroscopy Use of power tools for pedicle screw insertion Intrathecal morphine	*POD 0* Morphine PCA and IV diazepam Oral ketorolac as adjunctive Sit upright at the edge of bed Oral liquids if tolerated *POD 1* Shift onto oral analgesics Supervised mobilization out of bed Advance to soft and high fibre diet as per tolerance Bowel care regimen Urinary catheter removed if able to walk out of bed *POD 2–4* PCA discontinued completely Remove drains if used Independent short walks sit upright in chair Climb a flight of stairs if possible Encouraged for shower Discharge if Ambulating independently Tolerating oral intake and oral analgesics Passing flatus Periodic follow up with surgical, nursing and physiotherapy team

## Preoperative protocol

The co-operation of patients and parents with the medical team is required to successfully implement ERAS. Hence, patient and parental education about the expected protocol that will be followed, and the likely pain experience post-surgery becomes of utmost importance. Patient expectations are aligned with a structured physiotherapy program involving aerobic exercises as well as back strengthening and flexibility exercises for 6 weeks prior to surgery [18]. A dedicated scoliosis support group interacts with the patient so as to optimize the mental and emotional state of patients in preparation for surgery [15, 18, 19]. Patients are prescribed oral iron and multivitamin supplements to take daily for 1 month prior to surgery [18]. This is to cover any undiagnosed pre-existing vitamin deficiencies. Patients are also asked to wash either with a gentle soap or have a chlorhexidine wash on the night prior to surgery [17].

On the morning of surgery, patients are counseled about back care and the correct way of mobilization after surgery by a spinal physiotherapist [18]. Patients interact with the pain management nurse about the expected postoperative course that involves the anticipated trajectory of postoperative pain and ways to manage it as well as anticipated recovery [18, 19].

## Intraoperative protocol

ERAS protocols that were employed during surgery were directed towards reducing the surgical time and blood loss [18, 19]. Two studies also mentioned the specific protocol for use of anesthesia [18, 19]. Bolus dose of 50 mg/kg over 30 min of tranexamic acid given just before skin incision and then continued as 5 mg/kg/h for the duration of surgery [18, 19]. Intraoperative cell salvage is used to reduce the need for allogenic blood transfusion [18]. A bolus dose of intravenous (IV) antibiotics are administered at induction [18]. Isovolumic hemodilution is performed if considered appropriate by the anesthetist [19]. The patient is maintained on total intravenous anesthesia with propofol and remifentanil throughout surgery [19]. MAP is maintained between 55 to 80 mm Hg based on the phase of surgery [18]. SSEP and MEPs are performed during the procedure.

To reduce surgical time and blood loss, there was a common policy involving dual attending consultant surgeons [18]. To reduce the surgical time it was suggested to limit the fluoroscopy checks for verifying the placement of pedicle screws, but this of course needs to be balanced against patient safety [19]. It was also suggested to use power tools for placing the pedicle screws to reduce the surgical time [20]. Use of intra-thecal morphine injections (5 μg/kg up to 250 μg) along with subcutaneous bupivacaine wound infiltration at the end of surgery was utilized to enhance post-operative analgesia [15, 18, 19].

## Postoperative protocol

There are technical differences amongst various studies regarding postoperative protocols but the common aim was to restore the patient’s homeostasis early by managing pain efficiently, permitting early mobilization, and aiming for early discharge so as to reduce the length of hospital stay, avoid complications and reduce the burden on the health care system [12, 15, 18]. On the day of surgery, morphine Patient Controlled Analgesia (PCA) with IV diazepam was utilized for pain control and patients were encouraged to sit upright at the edge of the bed [14–17, 19]. Oral liquids were started as per patient’s tolerance. Oral Ketorolac was added as an adjunctive analgesic irrespective of the use of a drain if patients were able to tolerate it [16, 17, 19].

On the first post-operative day, patients were weaned off the PCA and oral analgesics commenced for management of pain. Patients were mobilized under the supervision of a physiotherapist out of bed as per pain tolerance once PCA was completely weaned off. If PCA was still continued, patients were encouraged to sit propped up to 30 degrees in bed. In the absence of any opioid-related nausea and vomiting, diet regimen was advanced to clear liquids and a high fiber diet if patients tolerated [11, 15, 17, 19]. All patients were placed on a bowel care regime so as to facilitate bowel movements. Once patients were able to get out of bed under supervision, the indwelling urinary catheter was removed and patients encouraged to walk to the bathroom for toileting needs [16–18]. Pain management nurses recorded pain scores on a daily basis to ensure adequate pain control [18, 19].

On post-operative day two until discharge, PCA was invariably discontinued and oral analgesics commenced as per the center specific pain management protocol [12–19]. There was conflicting literature on the use and duration of a drain [14, 18]. If utilized, all the sub-fascial drains were removed and patients encouraged to sit up in a chair and undertake independent short walks and if possible climb a flight of stairs [16, 19]. Patients were commenced on a general diet and encouraged to have a shower on the fourth post-operative day. Patients were discharged when they were ambulating independently, tolerating oral diet and oral analgesics without any nausea or vomiting and able to pass flatus [14, 18, 19]. Having bowel movements was not an essential criterion for discharge [18, 19]. After discharge, all patients were followed up periodically in outpatient clinics with the surgeon, specialty nurses, and physiotherapist and the progress documented. There was no clear guidance with regards to postoperative X-rays. These should be performed as per center-specific protocols at the discretion of the surgical team. Table 3 summarizes all of the measures for the ERAS protocol in the care of AIS patients undergoing posterior scoliosis correction.

## Discussion

This systematic review demonstrates that with the implementation of ERAS protocols in the care of patients undergoing posterior correction for AIS, there is a significant reduction in the length of stay without any significant increase in primary complication and readmission rates. Overall there was no significant difference between the two groups for operative time. However, two studies [12, 17] individually demonstrated operative time to be shorter in the ERAS group and one study [16] for the TP group. The use of navigation for inserting pedicle screws and Ponte’s osteotomy in more patients in the TP group was thought to be responsible by the authors for the increased surgical time in this group in the two studies [12, 17]. On the other hand, the use of navigation in the ERAS group added to surgical time in the study by Rao et al. [16]. There was no significant difference between the two groups for age, number of levels fused, preoperative magnitude of major curve and blood loss. It has been shown that correction of larger curves is associated with increased utilization of health care resources specifically the longer operative time and greater number of vertebral levels instrumented [22]. In this review, we found that both groups were comparable for pre-operative magnitude of curve and number of vertebral levels instrumented and hence these factors did not have any bearing on LOS between the two groups. Moreover, on regression analysis Fletcher et al. [17] did not find any correlation between the LOS, curve size or levels fused. This suggest that major determinants of LOS are unrelated to these intra-operative surgical factors.

The main feature that distinguished the ERAS from TP in all the studies was the use of multi-modal analgesia, early ambulation, and early removal of the drain and urinary catheter to ensure early discharge. Delayed mobilization and increased pain are major factors associated with delayed discharge and increased complication rates following posterior spinal fusion in patients with AIS. This affects the patient’s post-operative experience and also has financial implications for the health care system. Enhancements in treatment quality, and effectiveness are essential in improving perioperative care of patients undergoing posterior spinal fusion for AIS. Although substantial recent progress has been made in the surgical techniques, safety and efficacy, further work is required to improve patient-reported outcomes and minimize procedure-related morbidity. Despite the described incremental improvements, the average length of stay still remains 5.6 days in the USA for this subset of patients [23]. A patient-centered model that was proposed recently and bears resemblance to ERAS was of Perioperative Surgical Home (PSH). It is defined by the American Society of Anesthesiologists as “a patient-centered and physician-led multidisciplinary and team-based system of coordinated care that guides the patient throughout the entire surgical experience from the decision of need of an invasive procedure to discharge and beyond” [24]. This model aimed at standardization of perioperative care to reduce the errors that arise from inconsistency in care. Patient education, shared decision making, oriented customized recovery plan and performance targets were incorporated at each stage of patient care at PSH. Patients are followed by the pain team on a regular basis for the continuity of care. A coordinator nurse ensures the smooth transition of patients during all phases of patient care. An established perioperative surgical home has many elements of ERAS but the essence of this model is the co-ordination of all features of perioperative care rather than stringently implementing them [24]. Although this model is adopted by centers involved in the care of adult patients, its utility in the care of pediatric patients has yet to be reported [25].

Traditional postoperative care in patients undergoing PSF in AIS was often based on anecdotal protocols with variable outcomes. There is no uniformity in the literature for postoperative protocols for patient’s undergoing PSF for AIS. The need for a specific and uniform evidence-based protocol is important to enhance patient and process outcomes. With the use of multimodal analgesia and early enteral nutrition, ERAS improves the functional recovery of patients whilst improving post-operative pain scores. This is reflected by early mobilization, discharge, and reduced LOS observed across all the studies [25].

Another common element observed in all studies is the integration of multiple disciplines in the care of patients even before surgery. Kim et al. [19] highlighted that implementation of multifaceted care in the ERAS protocol was the single most statistically significant factor responsible for reduced LOS. The involvement of scoliosis support groups and the early introduction of patients to dedicated nurses and the pain management team played a vital role in the preparation and education of patients and parents about expectations in terms of surgery-related pain, its management, and mobilization regimen [27].

Although all the above-mentioned studies have different regimens for the management of postoperative pain, the use of multi-modal analgesia was a common concept. Early mobilization after surgery is one of the keystones of ERAS. Thus, pain scores might be expected to be higher in this protocol. In fact, the majority of studies showed that ERAS did not have an adverse effect on post-operative pain scores except for Sanders et al. [15] who reported the higher pain scores after the first postoperative day. This probably reflects the advantageous pre-conditioning of the patient with regards to pain and analgesic expectations.

It is important to consider adjuvant analgesics given the numerous side-effects of opioid analgesics, and their detrimental effect for early mobilization. A common finding in all the studies was the use of adjuvant analgesics like ketorolac combined with opioid analgesics. Although some authors have reported problems with Ketorolac and NSAIDs in major orthopedic surgery (e.g.. platelet dysfunction and interference with bone healing), there is growing evidence that ketorolac is safe in the pediatric orthopedic population and spinal fusions [14]. It also reduces the need and dosage of opioid intake, thus reducing related side effects and LOS [14].

Early mobilization and rehabilitation require active engagement of the patient during recovery from surgery. There is data demonstrating that early mobilization has positive impact on overall outcome after surgery [28]. Protocols adopting early mobilization have benefits of reduced medical complications, infections and a decrease in hospital stay.

Early oral intake after surgery improves the recovery of bowel function and is considered safe. This is commonly incorporated in rapid recovery protocols in other specialties [2]. Though there is no evidence of this in direct relation to spine surgery, early resumption of oral diet was a common feature in all ERAS protocols.

There was a significant reduction in the length of stay in patients undergoing ERAS when compared to conventional protocols. Lowest LOS associated with ERAS was noted in a study by Fletcher et al. [17]. In a retrospective analysis of 160 patients, the authors compared the LOS following accelerated discharge (AD) protocol at one center with the traditional discharge (TD) protocol at another center for patients undergoing PSF for AIS. 105 patients were included in the AD pathway while 45 patients were managed under TD pathway. The AD protocol focused on pre-operative patient and family education, minimizing the intraoperative blood loss, early weaning-off of PCA and transition to oral pain medications, early mobilization with physiotherapy, early resumption of oral liquids, and discharge irrespective of return of bowel function. There was a reduction by 48% in LOS in AD group (2.2 v/s 4.2 days, *p* < 0.0001). There was a significantly less total number of complications seen in AD group without any difference in rate of readmissions. Reduction in LOS was attributed to the utilization of a multi-specialty system-based pathway.

There was no significant difference between the rates of complications for the patients undergoing ERAS when compared to conventional protocols. The follow-up period for complications varied among the 7 papers included in the analysis of complication rates (from 30 days to 1 year). Complications related to surgical procedure (e.g. wound infection, malpositioned screw, failure of metal work) were taken as complications while readmission to hospital and visit to ED after surgery were included in the analysis of readmission rate. The lowest complication rates were seen in a study of Rao et al. [16]. In this retrospective analysis, 139 patients followed the ERAS protocol after undergoing posterior spinal fusion for AIS. This group had two separate subgroups based on different recovery protocols. However, since both these protocols were based ERAS principles, patients from both of these subgroups were pooled together in a single group. 1.4% (2/139) patients in the ERAS group had complications. One patient had a vocal cord palsy due to a misplaced pedicle screw. This improved completely after the removal and replacement of the pedicle screw. The second patient had a superficial wound infection which was treated with oral antibiotics. There was no significant difference between the readmission rates for the patients undergoing ERAS when compared to conventional protocol.

In a case–control retrospective analysis, Kim et al. [19] reported no readmissions among 36 patients following ERAS pathway. Muhly et al. [13] reported the second-lowest readmission rate in the ERAS group. In a retrospective analysis, after adaptation of rapid recovery protocols, the length of stay reduced from 5.7 days to 4 days without any significant increase in readmission rates (3.6% vs 2.9%). One patient was readmitted for analgesia-related nausea; 2 patients were admitted for evaluation of possible deep wound infections.

ERAS has a positive financial impact on the utilization of health care resources [27]. There are many factors that contribute to the overall cost of PSF in AIS patient with charges often exceeding $100,000 [8, 20]. Fletcher et al.[13] showed that when compared to traditional protocols, the use of ERAS protocols resulted in a 33% reduction in room charges ($1,885 ± 486 vs. $2,779 ± 617, *p* < 0.0001) and an 11% reduction in therapy charges ($554 ± 201 vs. $619 ± 219, *p* = 0.004). Sander’s et al. [15] showed that utilization of ERAS for the management of AIS patients undergoing PSF could reduce the postoperative hospital charges by 22% without an increase in complications and readmissions. Moreover, cost savings achieved by the implementation of ERAS often exceed the initial investment [30].

## Limitation

Nine of 10 studies in this review of literature were retrospective in nature and hence prone to selection bias. There are technical differences in the protocols followed in each study and for this reason, the random-effects model was used for the meta-analysis. There was a lack of heterogeneity of the interventions and recorded outcomes. Six out of the eight studies compared the complication rates between the ERAS and control groups; however, the definition of this outcome measure varied between the studies. This problem was also observed while comparing readmission rates between the two intervention groups as the thresholds for re-intervention may vary between centers and practices. Another limitation was that there was no record of delayed complications due to short follow-up, i.e. superficial/deep surgical site infection later requiring removal of metal work in any of the studies and we therefore are unable to conclude the impact of ERAS on delayed complications. Another problem with these studies was the lack of validated outcome measures for reporting patient satisfaction between the intervention groups. A robust, prospective multicentre RCT will provide definitive evidence of ERAS impact on patient outcomes and healthcare costs for PSF in AIS.

## Keypoints

- Implementation of ERAS in patients undergoing posterior spinal fusion for adolescent idiopathic scoliosis requires a patient-centered multi-disciplinary approach.
- Use of an ERAS protocol results in a significant reduction in length of stay as compared to traditional protocol.
- There was no significant difference between the two groups with regards to complications and readmission rates.
